# Potential Role of Social Distancing in Mitigating Spread of Coronavirus Disease, South Korea

**DOI:** 10.3201/eid2611.201099

**Published:** 2020-11

**Authors:** Sang Woo Park, Kaiyuan Sun, Cécile Viboud, Bryan T. Grenfell, Jonathan Dushoff

**Affiliations:** Princeton University, Princeton, New Jersey, USA (S.W. Park, B.T. Grenfell);; National Institutes of Health, Bethesda, Maryland, USA (K. Sun, C. Viboud, B.T. Grenfell);; McMaster University, Hamilton, Ontario, Canada (J. Dushoff)

**Keywords:** Coronavirus disease, COVID-19, SARS-CoV-2, severe acute respiratory syndrome coronavirus 2, respiratory diseases, zoonoses, viruses, pneumonia, social distancing, South Korea

## Abstract

In South Korea, the coronavirus disease outbreak peaked at the end of February and subsided in mid-March. We analyzed the likely roles of social distancing in reducing transmission. Our analysis indicated that although transmission might persist in some regions, epidemics can be suppressed with less extreme measures than those taken by China.

The first coronavirus disease (COVID-19) case in South Korea was confirmed on January 20, 2020 (*1*). In the city of Daegu, the disease spread rapidly within a church community after the city’s first case was reported on February 18 (*1*). Chains of transmission that began from this cluster distinguish the epidemic in South Korea from that in any other country. As of March 16, a total of 8,236 cases were confirmed, of which 61% were related to the church (*1*).

The Daegu Metropolitan Government implemented several measures to prevent the spread of COVID-19. On February 20, the Daegu Metropolitan Government recommended wearing masks in everyday life and staying indoors (*2*). On February 23, South Korea raised its national alert level to the highest level (*1*) and delayed the start of school semesters (*3*). Intensive testing and contact tracing enabled rapid identification and isolation of case-patients and reduction of onward transmission (*4*). We describe potential roles of social distancing in mitigating COVID-19 spread in South Korea by comparing metropolitan traffic data with transmission in 2 major cities.

## The Study

We analyzed epidemiologic data describing the COVID-19 outbreak in South Korea during January 20–March 16. We transcribed daily numbers of reported cases in each municipality from Korea Centers for Disease Control and Prevention (KCDC) press releases (*1*). We also transcribed partial line lists from press releases by KCDC and municipal governments. All data and code are stored in a publicly available GitHub repository (https://github.com/parksw3/Korea-analysis).

**Figure 1:**
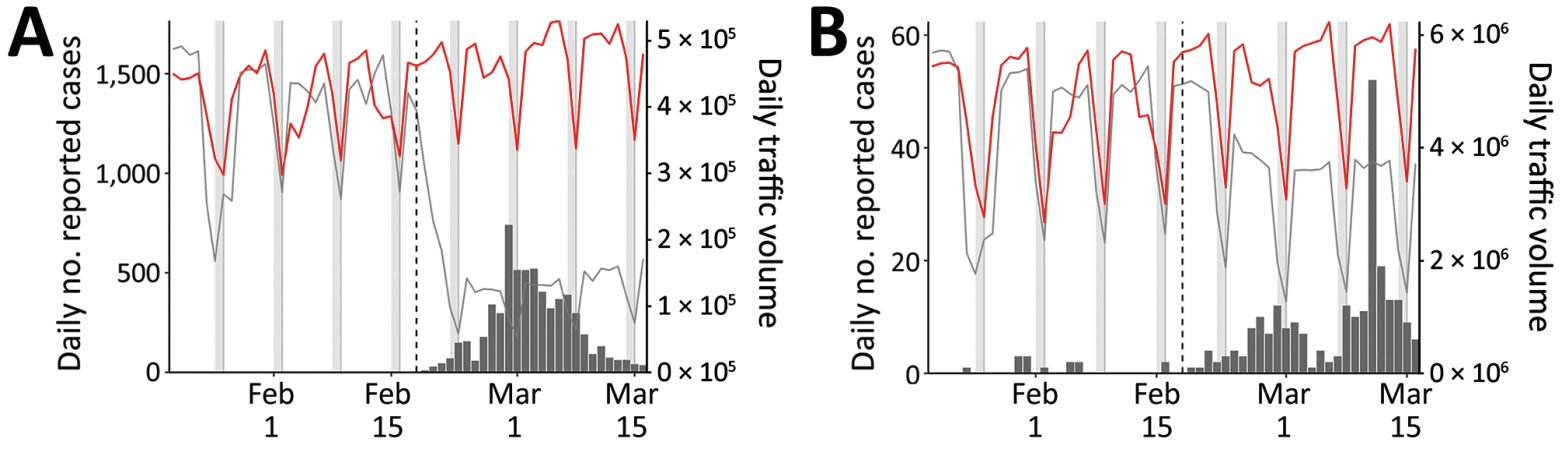
Comparison of daily epidemiologic and traffic data from Daegu (A) and Seoul (B) during the coronavirus disease (COVID-19) outbreak, South Korea. Black bars indicate no. COVID-19 cases; lines represent daily metropolitan traffic volume in 2020 (red) and mean daily metropolitan traffic volume during 2017–2019 (black). Daily traffic from previous years have been shifted by 1–3 days to align day of the weeks. Vertical dashed lines indicate February 18, 2020, when the first COVID-19 case was confirmed in Daegu. Gray bars indicate weekends.

We compared epidemiologic dynamics of COVID-19 from 2 major cities: Daegu (2020 population: 2.4 million) and Seoul (2020 population: 9.7 million). During January 20–March 16, KCDC reported 6,083 cases from Daegu and 248 from Seoul. The Daegu epidemic was characterized by a single large peak followed by a decrease (Figure 1, panel A); the Seoul epidemic comprised several small outbreaks (Figure 1, panel B).

We obtained the daily number of persons who boarded the subway or monorail in Daegu and Seoul during 2017–2020. For Daegu, we used data from https://data.go.kr for lines 1–3; for Seoul, we used data from https://data.seoul.go.kr for lines 1–9 (Figure 1). Soon after the first church-related case was reported, traffic volume decreased by »80% in Daegu and »50% in Seoul. To our knowledge, KCDC first recommended social distancing on February 29 (*1*), and no official guidelines existed regarding public transportation, which suggests that distancing was, at least in part, voluntary.

We reconstructed the time series of a proxy for incidence of infection *I_t_*, representing the number of persons who became infected at time *t* and reported later, and estimated the instantaneous reproduction number, R*_t_*, defined as the average number of secondary infections caused by an infected person, given conditions at time *t* (*5*). We adjusted the daily number of reported cases to account for changes in testing criteria and censoring bias (Appendix) and then sampled infection dates using inferred onset-to-confirmation delay distributions from the partial line list (Appendix) and previous estimated incubation period distribution (Table) to obtain our incidence proxy, *I_t_*. Finally, we estimated R*_t_* on the basis of the renewal equation (*5*):where *w_t_* is the generation-interval distribution randomly drawn from a prior distribution (Table). We weighted each sample of R*_t_* using a gamma probability distribution with a mean of 2.6 and a SD ± 2 to reflect prior knowledge (S. Abbott, unpub. data, https://doi.org/10.12688/wellcomeopenres.16006.1) and took weighted quantiles to calculate medians and associated 95% credible intervals. We estimated R*_t_* for February 2 (14 days after the first confirmed case) through March 10 (after which the effects of censoring were too strong for reliable estimates) (Appendix). All analyses were performed using R version 3.6.1 (https://www.r-project.org).

**Table:** Assumed incubation and generation-interval distributions in an analysis of the potential role of social distancing in mitigating the spread of coronavirus disease, South Korea, 2020*

Distribution	Parameterization	Priors	Source
Incubation period distribution	Gamma (*µ_I_, µ*^2^*_I_/σ*^2^)	*µ_I_* » gamma (6.5 d, 145); σ » gamma (2.6 d, 25)	(*6*)
Generation-interval distribution	Negative binomial (*µ_G_, θ*)	*µ_G_* » gamma (5 d, 62); θ » gamma (5, 20)	(*7**,**8*)

*Gamma distributions are parameterized using its mean and shape. Negative binomial distributions are parameterized using its mean and dispersion. Priors are chosen such that the 95% quantiles of prior means and standard deviations are consistent with previous estimates.

**Figure 2:**
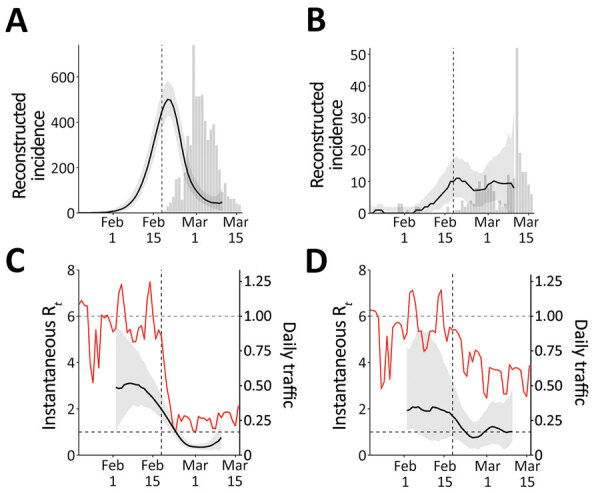
Comparison of reconstructed coronavirus disease incidence proxy and instantaneous reproduction number R*_t_* in Daegu (A, C) and Seoul (B, D), South Korea. The instantaneous reproduction number R*_t_* reflects transmission dynamics at time *t*. Black lines and gray shading represent the median estimates of reconstructed incidence (A, B) and R*_t_* (C, D) and their corresponding 95% credible intervals. Gray bars show the number of reported cases. Red lines represent the normalized traffic volume (daily traffic, 2020, divided by the mean daily traffic, 2017–2019). Vertical dashed lines indicate February 18, 2020, when the first case was confirmed in Daegu.

We reconstructed incidence proxy (Figure 2, panels A, B) and estimates of R*_t_* (Figure 2, panels C, D) in Daegu and Seoul. In Daegu, incidence peaked shortly after the first case was confirmed (Figure 2, panel A). In Daegu, symptoms had developed in the first case-patient on February 7; this person had visited the church on February 9 and 16, indicating the disease probably was spreading within the church community earlier (*1*). Likewise, the estimates of R*_t_* gradually decreased and eventually decreased to <1 approximately 1 week after the first case was reported, coinciding with the decrease in the metropolitan traffic volume (Figure 2, panel C). The initial decrease in R*_t_* was likely to have been caused by our resampling method for infection times for each reported case, which oversmooths the incidence curve and the R*_t_* estimates (K. Gostic, unpub. data, https://doi.org/10.1101/2020.06.18.20134858). In Seoul, estimates of R*_t_* decreased slightly but remained at »1 (Figure 2, panel D), which might be explained by less-intense social distancing. Stronger distancing or further intervention would have been necessary to reduce R*_t_* to <1 by March 10.

Although we found clear, positive correlations on a daily scale between normalized traffic and the median estimates of R*_t_* in Daegu (*r* = 0.93; 95% credible interval 0.86–0.96; Appendix) and Seoul (*r* = 0.76; 95% credible interval 0.60–0.87; Appendix), these correlations are conflated by time trends and by other measures that could have affected R*_t_*. We did not find clear signatures of lags in the correlation between R*_t_* and traffic volume (Appendix Figure 3). Patterns in R*_t_* were similar in directly adjacent provinces (Gyeongsangbuk-do and Gyeonggi-do), demonstrating the robustness of our analysis (Appendix Figure 4).

## Conclusions

The South Korea experience with COVID-19 provides evidence that epidemics can be suppressed with less extreme measures than those taken by China (*9*) and demonstrates the necessity of prompt identification and isolation of case-patients in preventing spread (*4*). Our analysis reveals the potential role of social distancing in assisting such efforts. Even though social distancing alone might not prevent spread, it can flatten the epidemic curve (compare Figure 2, panels B, D) and reduce the burden on the healthcare system (*10*).

Our study is not without limitations. Because of insufficient data, we could not account for differences in delay distributions or changes in testing capacity among cities; line list data were mostly derived from outside Daegu. Nonetheless, the sensitivity analyses support the robustness of our findings (Appendix Figures 5–8). We were unable to distinguish local and imported cases and thus might have overestimated R*_t_* (*11*). Conducting a separate analysis for Seoul that accounts for imported cases did not affect our qualitative conclusions (Appendix Figure 9). Finally, although the method of resampling infection time can capture qualitative changes in R*_t_*, estimates of R*_t_* can be oversmoothed and should be interpreted with care (K. Gostic, unpub. data, https://doi.org/10.1101/2020.06.18.20134858). Nonetheless, our estimates of R*_t_* are broadly consistent with previous estimates (*12*).

We used metropolitan traffic to quantify the degree of social distancing. The 80% decrease in traffic volume suggests that distancing measures in Daegu might be comparable to those in Wuhan, China (*13*). We were unable to directly estimate the effect of social distancing on population contacts or epidemiologic dynamics. Other measures, such as intensive testing and tracing of core transmission groups, are also likely to have affected transmission dynamics.

Our study highlights the importance of considering geographic heterogeneity in estimating epidemic potential. The sharp decrease in Daegu drove the number of reported cases in South Korea. Our analysis revealed that the epidemic remained close to the epidemic threshold in other regions, including Seoul and Gyeonggi-do. Relatively weak distancing might have assisted the recent resurgence of COVID-19 cases in Seoul (E. Shim, G. Chowell, unpub. data, https://doi.org/10.1101/2020.07.21.20158923).

Appendix Additional methods and results for analysis of the potential role of social distancing in mitigating spread of coronavirus disease, South Korea.
