# Supplementary material for: Potential Role of Social Distancing in Mitigating Spread of Coronavirus Disease, South Korea
Source: Emerg Infect Dis. 2020 Nov;26(11):2697–700. doi: 10.3201/eid2611.201099 (PMC7588540; doi:10.3201/eid2611.201099)
Supplement: Appendix — Additional methods and results for analysis of the potential role of social distancing in mitigating spread of coronavirus disease, South Korea. [file 20-1099-Techapp-s1.pdf]

Article DOI: https://doi.org/10.3201/eid2611.201099

# Potential Roles of Social Distancing in Mitigating Spread of Coronavirus Disease, South Korea

## Appendix

## Epidemiologic Data

The daily number of reported cases from each municipality was translated and transcribed from the KCDC press release (*1*). Following the KCDC's protocol, the daily number of reported cases before February 20, 2020, reflects the number of confirmed cases on each day. During February 21–March 1, 2020, the daily number of reported cases reflects the number of reported cases within the last 24 hours (9 a.m. to 9 a.m.). On March 2, 2020, the daily number of reported cases reflects the number of cases that were reported between 9 a.m. March 1, 2020, and 12 a.m. March 2, 2020. Since then, the daily number of reported cases reflects the number of reported cases within the last 24 hours (12 a.m. to 12 a.m.). The number of negative cases was not reported on January 25 and 31, 2020; we took the average of cumulative negative cases from 1 day before and after these dates instead to impute missing values. The daily number of reported cases by the KCDC may be slightly different from the reports by each municipal government as some cases may be transferred after they are confirmed. The sum of daily number of reported cases by the KCDC may be also slightly different from the cumulative number of cases reported the KCDC because it does not reflect possible location changes of the confirmed cases after reporting.

## Reconstruction of Incidence Time Series

According to the KCDC press release (*1*), testing criteria expanded 4 times during January 20–March 16, 2020: January 28, February 7, February 20, and March 2, 2020. We accounted for these changes by assuming that the proportion positive should remain roughly constant if we follow a consistent protocol of identifying and deciding whom to test. To do so,

we calculated the relative proportion of positive cases during each period (divided by the between-period mean) and multiplied the daily number of reported cases by the relative proportions of the corresponding criterion. Sensitivity analyses showed that results are robust to these adjustments (Appendix Figures 5–8).

We then estimated time-dependent *backward* onset-to-confirmation delay distributions from the partial line list: Given a cohort of infected individuals who were confirmed on the same day, what is the probability distribution of the onset-to-confirmation delay? The backward delay distribution depends on changes in the number of symptomatic cases—e.g., when the number of symptomatic cases is increasing, the backward delay distribution is likely to be shorter because individuals are more likely to have developed symptoms recently. The backward delay distribution was inferred using a negative-binomial regression with log-link using the brms package (*2*). Time-dependent mean of the negative binomial distribution is modeled using splines. We assumed weakly informative priors on the fixed effects: normal distributions with mean of 0 and standard deviation of 2; note that these distributions are priors on link scale.

For each posterior sample of the backward delay distribution, we drew a random sample of onset-to-confirmation delay and incubation period for each confirmed case. This allowed us to obtain posterior samples of possible infection dates for each case, which were then converted into posterior samples of incidence time series.

To account for right-censoring in the reported cases, we also estimated time-dependent *forward* onset-to-confirmation delay distribution using the same negative-binomial regression model: Given a cohort of infected individuals who became symptomatic on the same day, what is the probability distribution of the onset-to-confirmation delay? The forward delay distribution reflects the changes in the accuracy of case identification—e.g., a decrease in the delay reflects improvement in accuracy.

To estimate the forward delay distribution, we modified the stan code from the negative-binomial regression that we used to infer the backward delay distribution to account for right-censoring (in the observed delays) and ran the code using the RStan package (*3*). In particular, we modified the likelihood of the negative-binomial regression such that given a delay of $x_i$ days, symptom onset day $t_i$ and the day of measurement of $t_{max}$, the likelihood of observing the delay is given by:

$$\frac{f(x_i|\mu(t_i),\theta)}{F(t_{max} - t_i|\mu(t_i),\theta)},$$

where $f$ is the negative binomial distribution with time-dependent mean $\mu(t_i)$ and dispersion parameter $\theta$. This likelihood accounts for the fact that the delay between symptom onset and confirmation cannot be longer than $t_{max} - t_i$ (otherwise, the case will be reported after $t_{max}$). Convergence is assessed by the lack of warning messages from the RStan package (*3*).

For each combination of date of infection and a posterior sample of the forward delay distribution, we drew 1,000 samples of incubation periods and onset-to-confirmation delays and calculated the median probability that an individual infected on a given day will be confirmed before March 16, 2020. Finally, we divided the daily number of infected cases by the median probability this probability. We used the reconstructed time series of incidence proxy to estimate $\mathcal{R}_t$.

**References**


1. Korea Centers for Disease Control and Prevention (KCDC). Press release [in Korean] [cited 2020 Jan 20–Jul 23]. Korea Centers for Disease Control and Prevention). Press release [in Korean]. Release dates 2020 Jan 20–Mar 16 [cited 2020 Jan 20–Jul 23]. https://www.cdc.go.kr/board/board.es?mid=a20501000000&bid=0015#

2. Bürkner P. brms: an R package for Bayesian multilevel models using Stan. J Stat Softw. 2017;80:1–28. https://doi.org/10.18637/jss.v080.i01

3. Stan Development Team. RStan: the R interface to Stan. R package version 2.19.3. 2020 [cited 2020 Jul 28]. https://cran.r-project.org/web/packages/rstan/vignettes/rstan.html

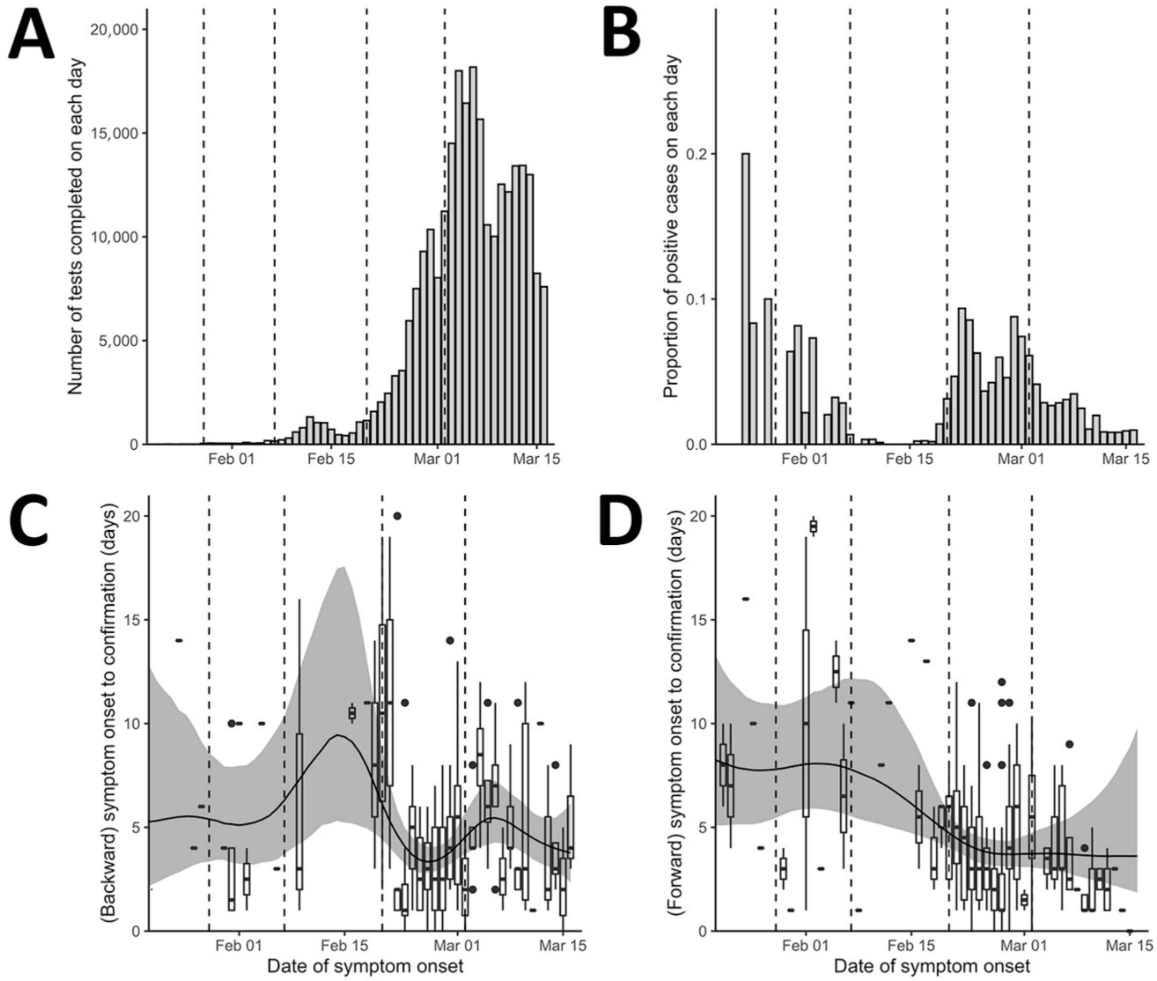


**Appendix Figure 1.** Changes in the number of tests and delay distributions over time. Vertical lines indicate the date on which testing criteria expanded. Box plots (C, D) represent the observed delays. Black lines and gray ribbons represent the median estimates of the mean delays and their associated 95% credible intervals.

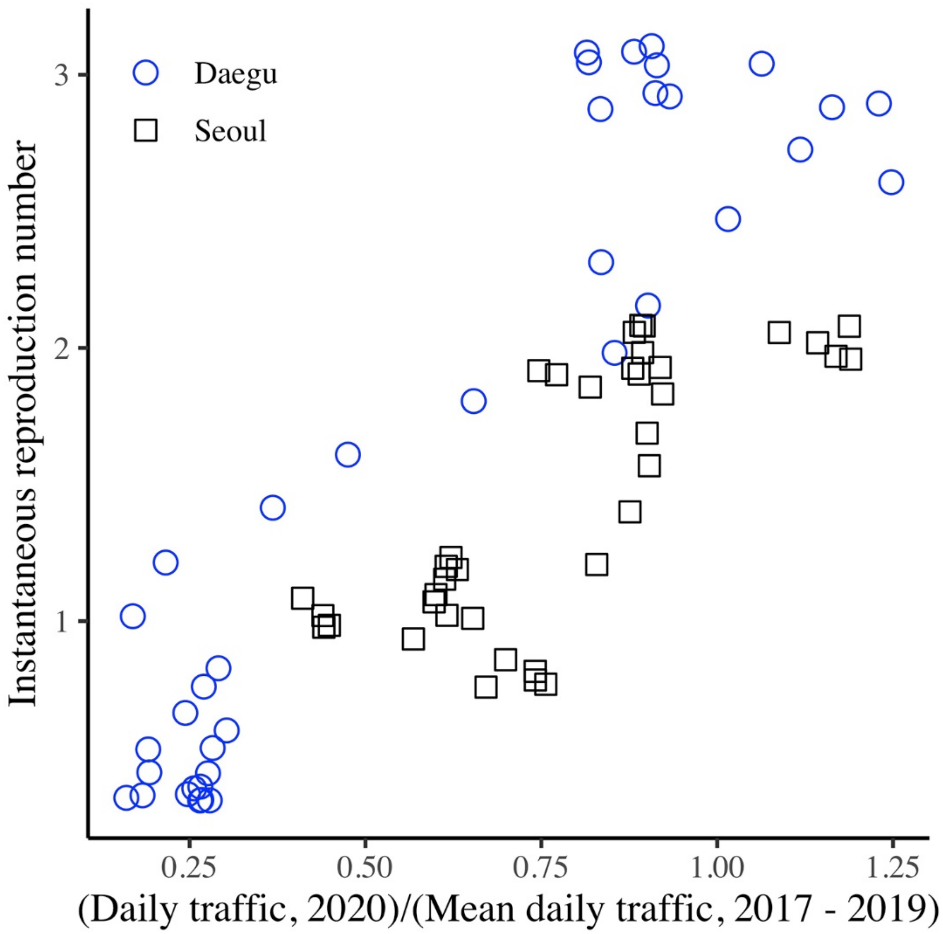


**Appendix Figure 2.** Scatter plot of the normalized traffic volume and the median estimates of $\mathcal{R}_t$ on a daily scale.

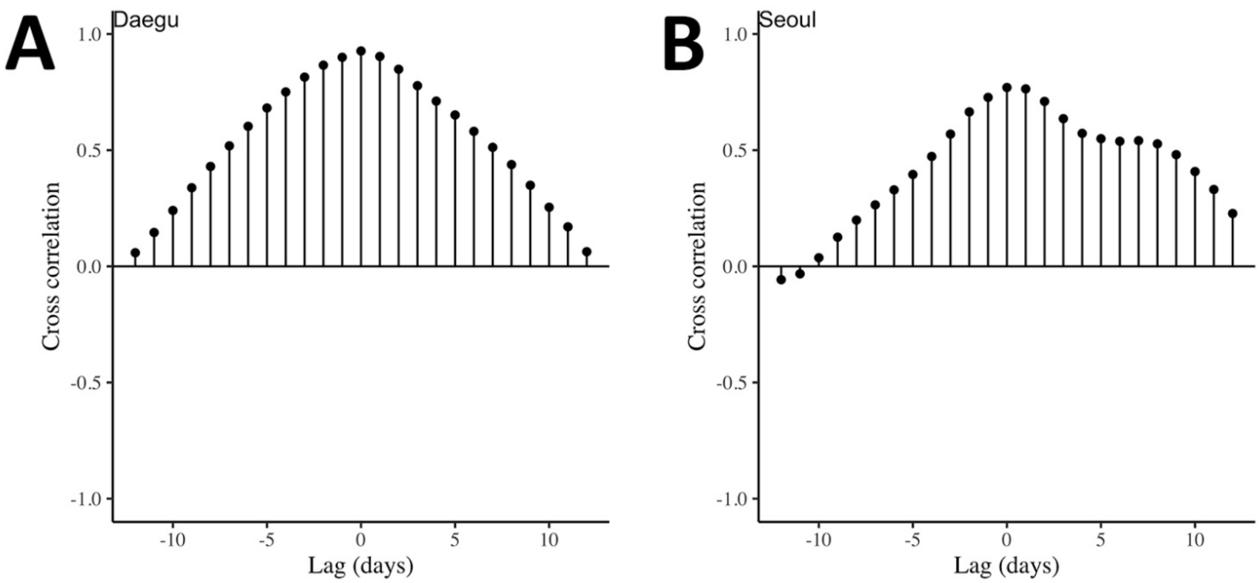


**Appendix Figure 3.** Cross correlation between the normalized traffic volume and the median estimates of $\mathcal{R}_t$ in Daegu (A) and Seoul (B).

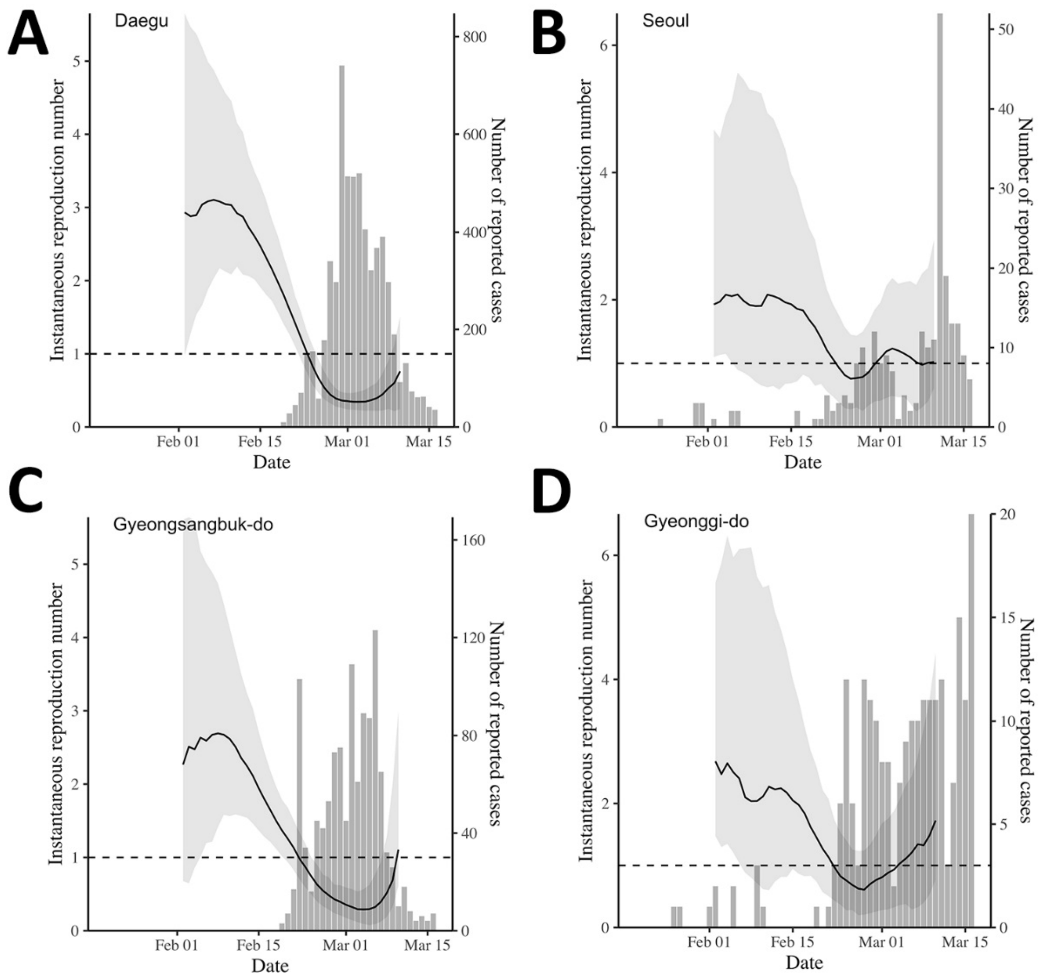


**Appendix Figure 4.** Comparison of $\mathcal{R}_t$ estimates and the daily number of reported cases in Daegu (A), Seoul (B), Gyeongsangbuk-do (C), and Gyeonggi-do (D).

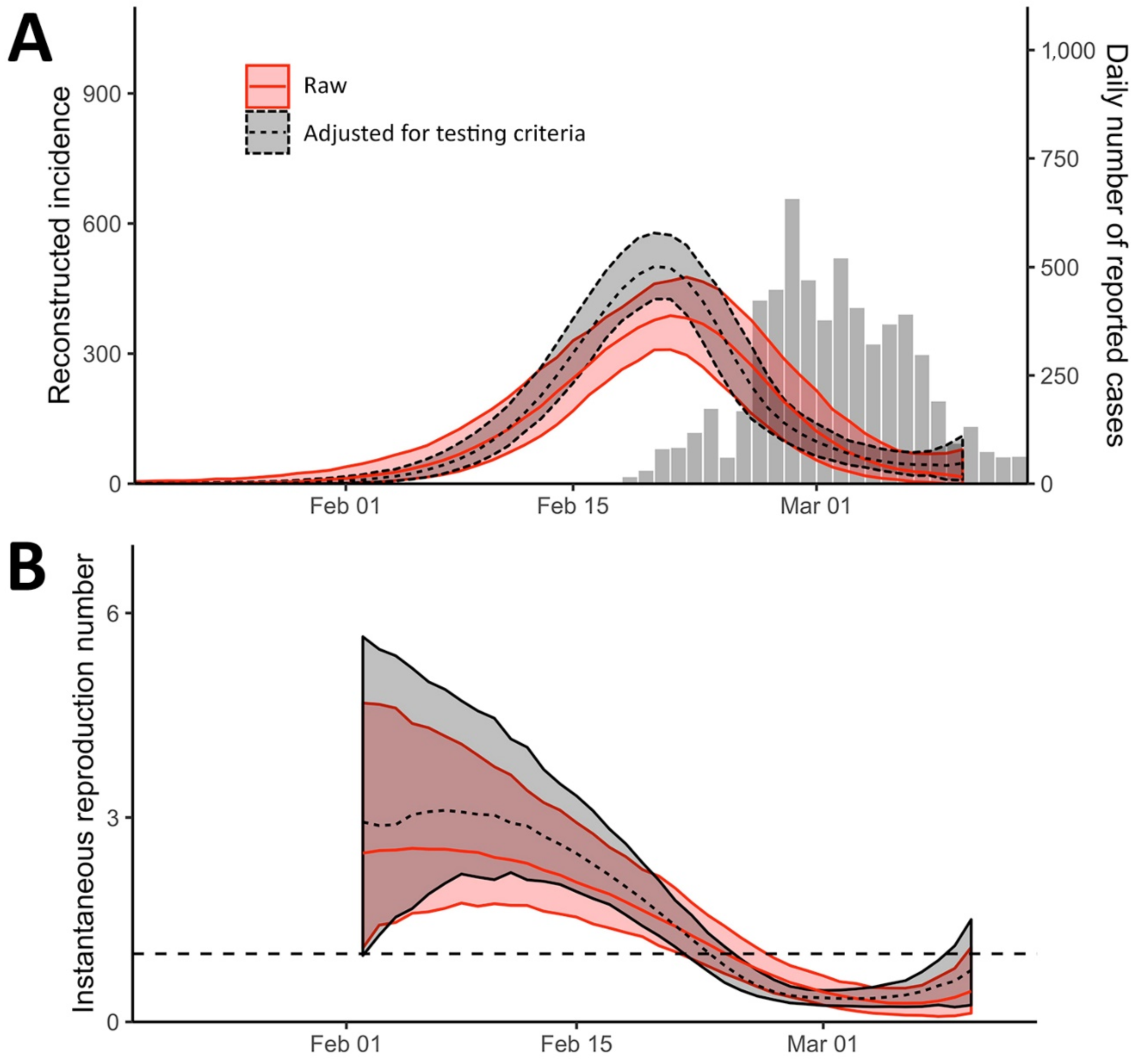


**Appendix Figure 5.** Sensitivity analysis of $\mathcal{R}_t$ estimates in Daegu with respect to changes in testing criteria.

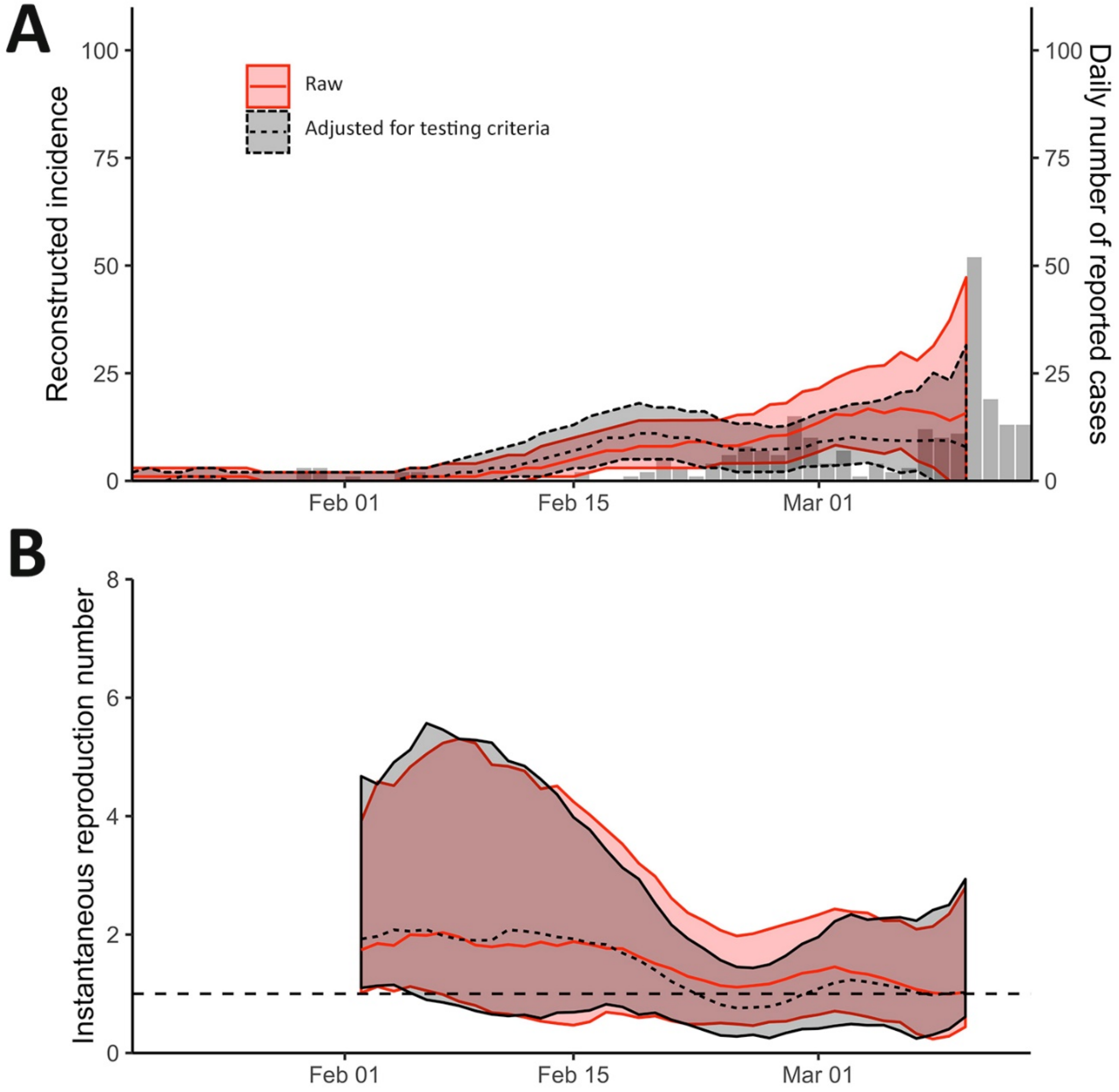


**Appendix Figure 6.** Sensitivity analysis of $\mathcal{R}_t$ estimates in Seoul with respect to changes in testing criteria.

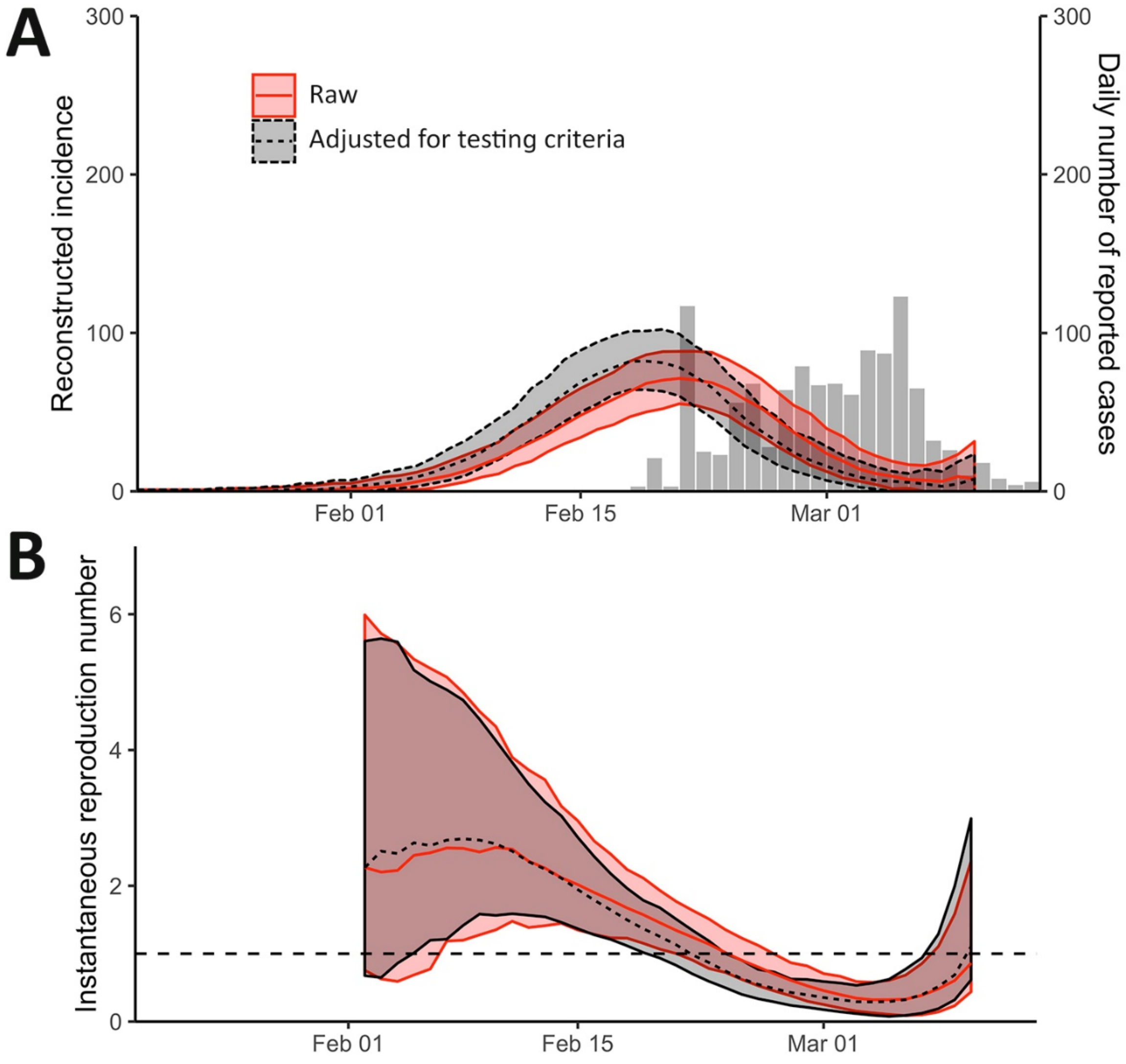


**Appendix Figure 7.** Sensitivity analysis of $\mathcal{R}_t$ estimates in Gyeongsangbuk-do with respect to changes in testing criteria.

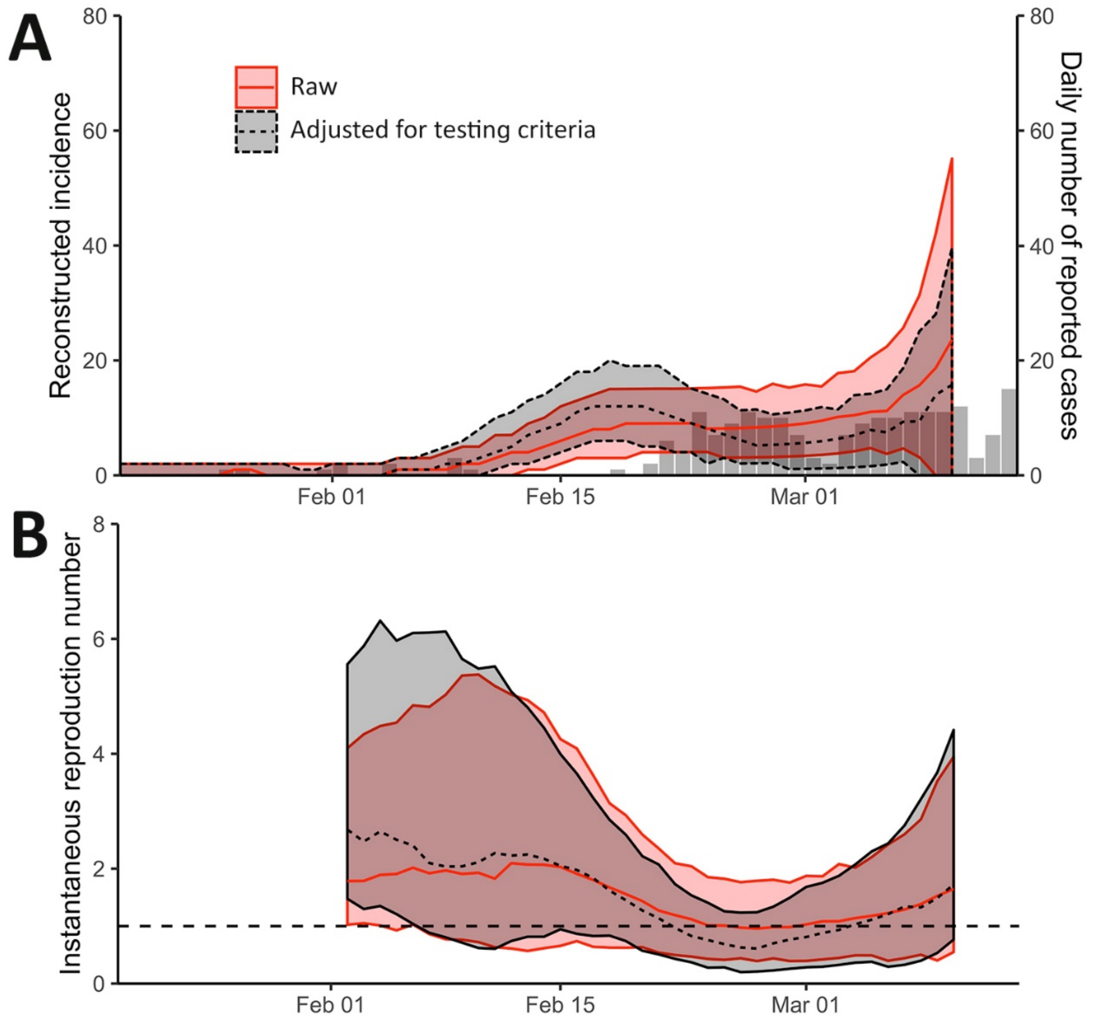


**Appendix Figure 8.** Sensitivity analysis of $\mathcal{R}_t$ estimates in Gyeonggi-do with respect to changes in testing criteria.

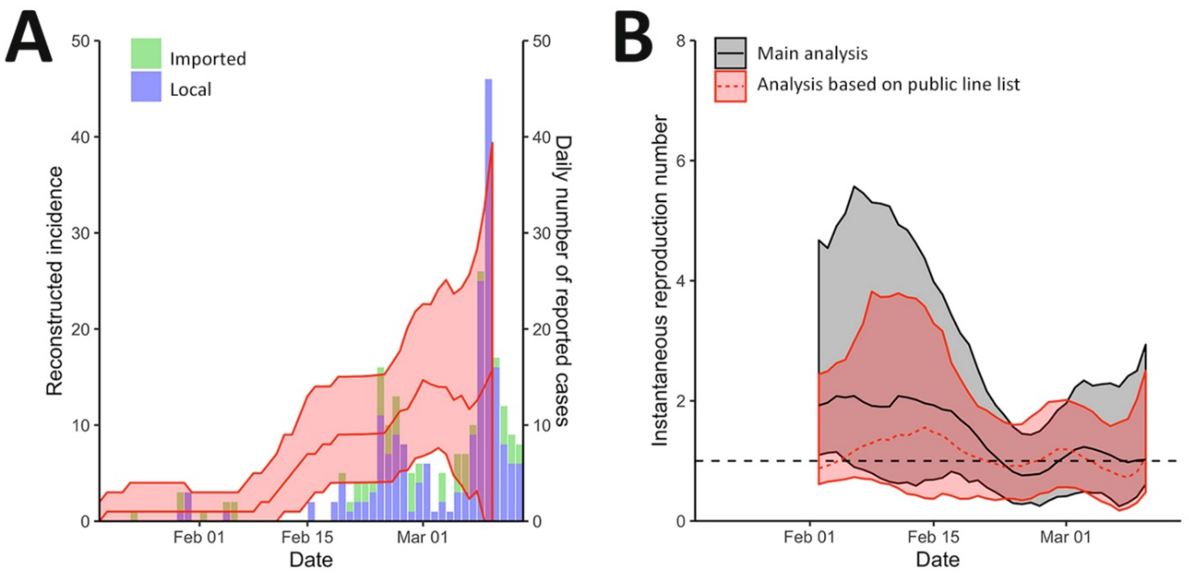


**Appendix Figure 9.** Comparison $\mathcal{R}_t$ in Seoul using the number of reported cases by the KCDC and public line list provided by the Seoul Metropolitan Government. Using public line list, we reconstructed incidence for local $I_t^{local}$ and imported $I_t^{imported}$ cases separately based on the method described in the main text. Then, we estimated the time-dependent reproduction number via $\mathcal{R}_t = I_t^{local} / \sum_{k=1}^{14} I_{t-k} w_k$, where $I_t = I_t^{local} + I_t^{imported}$. We did not account for changes in testing criteria in this analysis. The line list was obtained from http://news.seoul.go.kr/welfare/archives/513105 and https://www.seoul.go.kr/coronaV/coronaStatus.do.
